# Novel restriction factor RNA-associated early-stage anti-viral factor (REAF) inhibits human and simian immunodeficiency viruses

**DOI:** 10.1186/1742-4690-11-3

**Published:** 2014-01-10

**Authors:** Kelly M Marno, Babatunji W Ogunkolade, Corinna Pade, Nidia MM Oliveira, Eithne O’Sullivan, Áine McKnight

**Affiliations:** 1 Centre for Immunology and Infectious Disease, Blizard Institute, Barts and The London School of Medicine and Dentistry, Queen Mary University of London, London, UK

## Abstract

**Background:**

The discovery of novel anti-viral restriction factors illuminates unknown aspects of innate sensing and immunity. We identified RNA-associated Early-stage Anti-viral Factor (REAF) using a whole genome siRNA screen for restriction factors to human immunodeficiency virus (HIV) that act in the early phase of viral replication.

**Results:**

We observed more than 50 fold rescue of HIV-1 infection, using a focus forming unit (FFU) assay, following knockdown of REAF by specific siRNA. Quantitative PCR was used to show that REAF knockdown results in an increase of early and late reverse transcripts which impacts the level of integration. REAF thus appears to act at an early stage of the viral life cycle during reverse transcription. Conversely when REAF is over-expressed in target cells less infected cells are detectable and fewer reverse transcripts are produced. Human REAF can also inhibit HIV-2 and simian immunodeficiency virus (SIV) infection. REAF associates with viral nucleic acids and may act to prevent reverse transcription.

**Conclusions:**

This report firmly places REAF alongside APOBECs and SAMHD1 as a potent inhibitor of HIV replication acting early in the replication cycle, just after cell entry. We propose that REAF is part of an anti-viral surveillance system destroying incoming retroviruses. This novel mechanism could apply to invasion of cells by any intracellular pathogen.

## Background

The extent of the cellular armoury against viral infection is becoming increasingly appreciated. In particular human immunodeficiency virus (HIV) must overcome many cellular obstacles on its replication pathway to the nucleus. Once HIV enters the cytoplasm its genomic RNA is reverse transcribed by the virally encoded reverse transcriptase (RT), resulting in hybrid RNA:DNA intermediates. The RNase H activity of RT degrades the RNA from these hybrids resulting in single stranded (ss)DNA from which the second DNA strand is synthesised. Immediately upon initiation the process of reverse transcription is susceptible to members of the apolipoprotein B mRNA-editing, enzyme-catalytic, polypeptide-like (APOBEC) family by inducing deoxycytidine to deoxyuridine mutations in the nascent DNA [1]. In primary macrophages, dendritic cells and resting CD4^+^ T cells SAMHD1 degrades dNTPs which are required for efficient reverse transcription [2-4]. Two other factors p21 and PAF1 act at an early stage however their mechanism of action is not yet understood [5,6]. Once reverse transcription is complete the pre-integration complex (PIC) containing the double stranded (ds)DNA is then formed and integrated into the genome of the host cell. This process is inhibited by the TRIM28 (KAP1)/SETDB1 complex [6,7]. Once the provirus is integrated the late phase of the replication cycle begins with the production of viral proteins [8]. Finally, a plasma membrane located restriction factor tetherin/BST-2/CD317, prevents viruses from leaving the cell at the late budding stage of the life cycle [9].

Using a whole genome siRNA screen [6], we identified RPRD2 (here called RNA-associated Early-stage Anti-viral Factor; REAF) as a potential restriction factor. REAF is a protein of previously unknown function. Our data suggest that it acts at an early post-entry stage of HIV-1 replication, during or following the initiation of reverse transcription.

Characterising the action of a novel restriction factor reveals molecular details of HIV replication and innate host immunity and more importantly may lead to novel therapeutics for HIV and possibly other viral infections.

## Results

### REAF inhibits HIV and SIV infection and occurs at an early post-entry stage of replication

**Figure 1:**
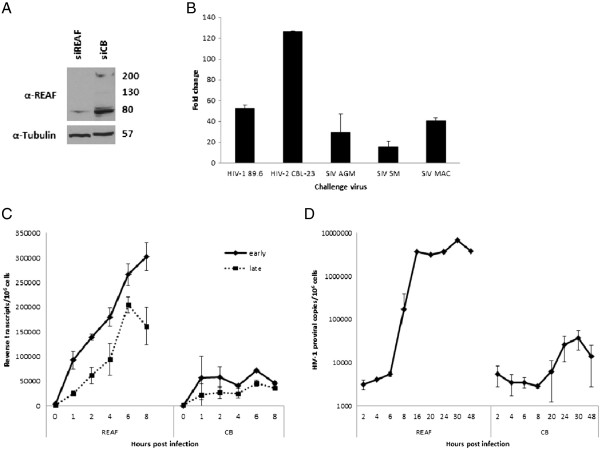
**REAF inhibits HIV and SIV infection and occurs at an early post-entry stage of replication. (A)** Western blot of HeLa-CD4 cell lysate following REAF siRNA knockdown compared with CB control. REAF is shown as three bands at 80, 130 (faint) and 220 kDa, but not all bands are detectable in all experiments. Tubulin (57 kDa) is added as a loading control. **(B)** 72 hr post siRNA knockdown of REAF, HeLa-CD4 cells are challenged with HIV-1^89.6^, HIV-2^CBL-23^, SIV^AGM^ (African Green Monkey; TYO-1), SIV^MAC^ (Macaque; 32H) and SIV^SM^ (Sooty Mangabey; B670). p24 immunostaining 48 hr post infection (pi) indicates that REAF rescues viral replication. Results are shown as fold change compared with a non-targeting control siRNA (CB). **(C)** siRNA knockdown of REAF results in enhanced HIV-1 RT products – early and late (0–8 hr pi) and **(D)** proviral DNA (*Alu*-*gag*, 2–48 hr pi). HIV-1 DNA copies are normalised to genomic GAPDH and presented per million cells. Results are mean ± s.d. of a representative experiment performed in duplicate.

The full details of our genome wide siRNA screen that identified new cellular anti-viral restriction factors acting at the early stages of HIV-1 replication have previously been published [6]. Briefly, HeLa-CD4 cells were transfected with an siRNA library targeting 19,121 human genes and then challenged with an HIV-1^89.6R^ pseudovirus carrying a GFP reporter gene (HIV-1 *gag/pol/tat* and *rev*, HIV-2 MCR Env). Gene knockdowns resulting in enhanced viral replication were chosen for further validation. For REAF validation, 4 siRNAs targeting different regions of REAF mRNA were initially tested to eliminate the possibility of off-target effects. 3/4 siRNAs showed the same phenotype of rescue (Additional file 1). A single siRNA was chosen for all subsequent experiments requiring silencing of REAF (siRNA REAF 4; Figure 1A, target sequence in Additional file 1).

To quantify the ability of REAF to rescue infection, HeLa-CD4 cells were challenged with dual tropic wild type HIV-1^89.6^ following siRNA knockdown. HIV-1 infection was strongly rescued following REAF silencing (>50 fold); from 6.9 × 10^2^ to 3.5 × 10^4^ focus forming units (FFU)/ml (Fold change cf. CB control; Figure 1B, Raw FFU data; Additional file 2). To determine if this anti-viral effect was restricted to HIV-1 we also tested for activity against HIV-2^CBL-23^ and various strains of simian immunodeficiency virus (SIV); African Green Monkey (TYO-1, SIV^AGM^), Macaque (32H, SIV^MAC^) and Sooty Mangabey (B670, SIV^SM^). We observed significant rescue of all viruses between 15–126 fold (Fold change cf. CB control; Figure 1B, Raw FFU data; Additional file 2).

There are multiple stages in the virus life cycle vulnerable to attack by cellular restriction factors. To gain insight into the action of REAF anti-viral activity we determined the point in the viral life cycle which is impeded. Cells were challenged with HIV-1^89.6^ after REAF was down modulated. Using real time quantitative PCR (qPCR) the initiation and completion of negative strand strong-stop DNA (−sssDNA; early) and full length (late) RT products were measured at various time points after infection (Figure 1C). Knockdown of REAF enhanced both early and late transcript levels within 1–2 hours of viral challenge, indicating that the reverse transcription process is targeted. An *Alu*-PCR assay subsequently found an increase in the amount of detectable integrated provirus (Figure 1D). This is probably a result of more available full length viral cDNA transcripts and correlates with a block by REAF during the reverse transcription process.

### Over-expression of REAF confers restriction which is viral route of entry dependent

**Figure 2:**
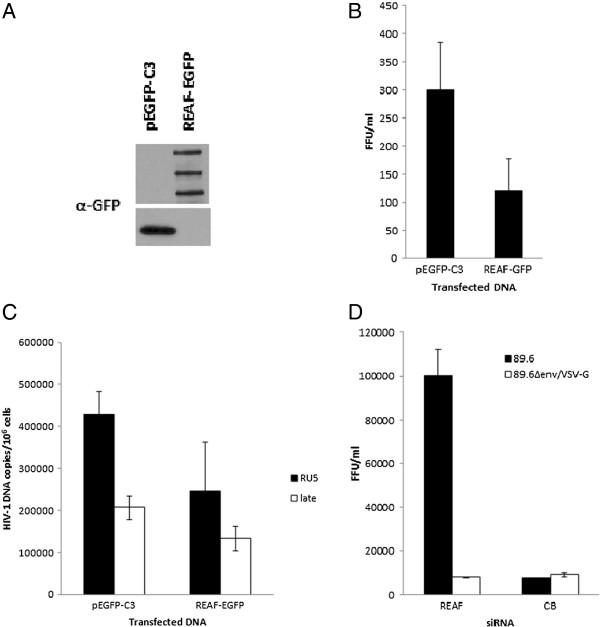
**Over-expression of REAF confers restriction which is viral route of entry dependent. (A)** Over-expression of REAF restricts early HIV-1 DNA production. HEK 293 T cells were transiently transfected with pEGFP-C3 or REAF-EGFP and the Western blot probed with α-GFP antibody. Transfection of REAF-EGFP results in expression of REAF (110, 160 and 250 kDa including EGFP tag (30 kDa). Endogenous REAF was knocked out of HeLa-CD4 cells using siRNA targeting its 3’ UTR. **(B)** Infected cells were measured following transient over-expression of REAF-EGFP or pEGFP-C3 and challenge with HIV-1^89.6^. Results are shown as FFU/ml and are mean ± s.d. of a representative experiment performed in duplicate. **(C)** Transient over-expression of REAF-EGFP in HeLa-CD4 cells results in decreased RU5 and late RT products 8 hr post challenge with HIV-1^89.6^. HIV-1 DNA copies are normalised to genomic GAPDH and presented per million cells. Results are mean ± s.d. of a representative experiment performed in duplicate. **(D)** Restriction by REAF is dependent upon viral route of entry. HIV-1^89.6Δenv^ was pseudotyped with VSV-G envelope and used to challenge HeLa-CD4 cells following REAF siRNA knockdown. p24 immunostaining was used to detect FFU compared with HIV-1^89.6^. Results are shown as FFU/ml and are mean ± s.d. of a representative experiment performed in duplicate.

To confirm that REAF protein is responsible for the rescue of viral replication observed following siRNA knockdown, over-expression experiments were also performed. To reconstitute the restriction, an EGFP-tagged expression construct for REAF was generated. Over-expression of REAF-EGFP was confirmed by Western blot analysis (Figure 2A). Similar to endogenous REAF protein (Figure 1A) the REAF-EGFP expression construct also produced three bands. As expected, the over-expression of REAF-EGFP was able to inhibit viral replication following challenge with HIV-1^89.6^. In contrast to other REAF siRNA knockdown experiments, here HeLa-CD4 cells were pre-treated with siRNA targeting the 3’ untranslated region (UTR) of REAF in order to degrade endogenous REAF before transfection of REAF-EGFP and pEGFP-C3 (empty vector) expression constructs (Western blot and target sequence data for REAF 3’UTR siRNA shown in Additional file 3). The inhibition by exogenous REAF in these experiments is weak compared to the >50 fold increase observed following siRNA knockdown. This is due to only poor (<10%) transfection efficiencies being achieved given the large size of the REAF-EGFP construct (Additional file 4). In spite of these technical difficulties, we were able to observe an inhibition of viral replication with REAF-EGFP over-expression by p24 immunostaining (Figure 2B). To confirm this inhibition, the levels of HIV-1^89.6^ DNA were measured in HeLa-CD4 cells similarly treated with REAF 3’UTR siRNA. After transfection of REAF-EGFP and challenge with HIV-1^89.6^, the amount of RU5 and late reverse transcripts was measured 8 hr pi, showing a 1.7 and 1.5 fold decrease respectively in cells expressing REAF-EGFP compared with pEGFP-C3 alone (Figure 2C). Given the poor transfection efficiency described above, these observations correlate with the qPCR results following siRNA knockdown of REAF, where a 4.5 fold increase in late transcripts is measured at the same time point (Figure 1C).

We previously reported that the Lv2 post entry restriction to HIV can be dependent on the envelope mediated route of entry [10-13]. To determine whether the anti-viral activity of REAF is route of entry dependent, HIV-1^89.6Δenv^/VSV-G pseudotyped virus was used to challenge HeLa-CD4 cells following REAF knockdown by siRNA. The 89.6Δenv construct contains the full length 89.6 genome, with a stop codon in the Env ORF, so these pseudotyped virions still produce p24 following integration and thus infection was measured by p24 immunostaining as above. In contrast to HIV-1^89.6^, no rescue of infected foci was observed after challenge with HIV-1^89.6Δenv^/VSV-G pseudotypes (Figure 2D). VSV-G triggers entry by clathrin-mediated endocytosis through a ubiquitously expressed glycolipid. These results suggest that REAF-mediated restriction of HIV-1^89.6^ is circumvented by a VSV-G mediated route of entry.

### REAF interacts with viral nucleic acids

**Figure 3:**
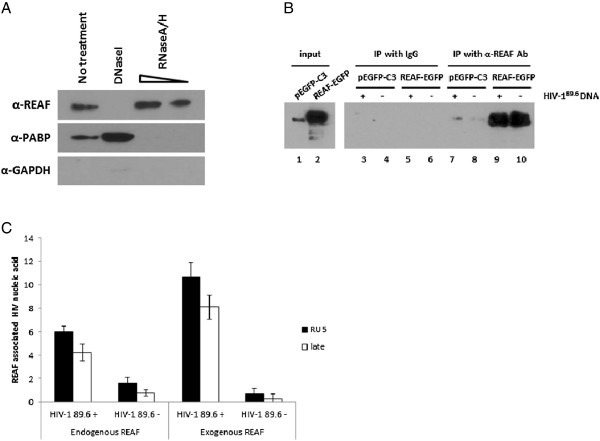
**REAF interacts with viral nucleic acids. (A)** Oligo d(T) immunoprecipitation of HeLa-CD4 cell lysate shows that REAF associates with captured RNA. Cell lysate was treated with DNase or titrated RNaseA/H before incubation with oligo (dT) beads. Immunoprecipitated protein was analysed by Western blotting and probed for REAF along with positive (PABP) and negative (GAPDH) controls. **(B)** HEK 293 T cells were transiently transfected with pEGFP-C3 or REAF-EGFP and the Western blot probed with α-REAF antibody. Endogenous and exogenous REAF are detectable in input samples (lanes 1 and 2) and following IP of samples transfected with pEGFP-C3 (endogenous REAF; lanes 7 and 8) or REAF-EGFP (endogenous and exogenous REAF; lane 9 and 10) with α-REAF antibody, but not after IP with IgG alone (lanes 3–6). **(C)** The amount of RU5 or late HIV-1 DNA qPCR product is quantified and normalised to input and IgG negative controls. Endogenous REAF associates with viral nucleic acids and this is enriched in the cells over-expressing REAF-EGFP.

As stated above, the function of REAF is previously unknown thus giving no clues as to its possible mode of interaction with viral replication. The timing of its inhibition during the reverse transcription phase of the viral life cycle suggested that REAF may interact with nucleic acids. To investigate this possibility, and determine if endogenous REAF associates with RNA, immunoprecipitation using oligo (dT) magnetic beads was performed. Oligo (dT) beads were incubated with HeLa-CD4 lysate prior to isolation of the bead-bound RNA/protein. As controls, cell lysates were additionally treated with either DNase or a titration of an RNaseA/RNaseH cocktail before the beads were added. The immunoprecipitated protein was then analysed by Western blotting for the presence of REAF. Results for the untreated sample show that REAF protein was specifically precipitated when oligo (dT) was used as bait (Figure 3A). Following DNase treatment, REAF is no longer detected suggesting that REAF may associate with complexes containing DNA. RNaseA specifically cleaves ssRNA, while RNaseH degrades DNA:RNA hybrid complexes. REAF was still found to be associated with the oligo (dT) beads in samples treated with RNase, and surprisingly the amount of REAF appeared to increase at higher concentrations of these enzymes. This may suggest that as more RNA is degraded, additional binding sites for REAF are revealed, resulting in the increased association observed. It could be argued that the REAF binding observed was due to the protein binding directly to the beads used. To eliminate this samples were also analysed for a positive control (poly(A) binding protein; PABP) which binds to the poly(A) tail of mRNA. As expected this protein is no longer detected in the RNase treated samples confirming that REAF binds through an RNA bridge and not directly to the beads themselves. It is also unlikely that the loss of REAF binding in the DNase treated samples is due to digest of oligo (dT) by DNase, as the enzyme is physically removed from the lysate before addition of the oligo (dT) beads. The success of this removal was confirmed by the presence of PABP in DNase treated samples. The negative control (GAPDH) confirms the oligo(dT) beads are binding specifically to proteins associated with nucleic acids. Thus the results showing that REAF associates (directly or indirectly) with polyadenylated RNA and DNA are compatible with our hypothesis that it can potentially target incoming viral genomes or RNA:DNA intermediates or double stranded (ds)DNA products of reverse transcription.

To investigate this further, HEK 293 T cells were transfected with vectors expressing either REAF-EGFP or pEGFP-C3 along with the HIV-1^89.6^ molecular clone DNA. All samples were lysed and incubated with either rabbit IgG (control) or α-REAF antibody conjugated protein A/G beads before immunoprecipitation (IP). Specific isolation of REAF protein by IP was confirmed by Western (Figure 3B). Following extensive washing, total RNA was isolated from the bead-bound complexes and reverse transcription (RT)-qPCR, using viral specific primers (RU5 and late), was performed. All results were normalised to input and the negligible level of qPCR products detected in IgG IP samples. Following this analysis, in cells only expressing endogenous REAF (transfected with pEGFP-C3), a 3.8 and 5.5 fold enrichment of viral RU5 and late transcripts respectively was measured compared with samples that were not transfected with virus (first half of Figure 3C). A further enrichment in both RU5 (16 fold) and late (32 fold) viral cDNA was seen following over-expression of REAF-EGFP (second half of Figure 3C), indicating a specific association of REAF protein and viral nucleic acids. To confirm the RT-qPCR results, a standard PCR was performed on the isolated cDNA and terminated at a non-saturating number of amplification cycles. These PCR products were run on a 4% agarose gel and visually confirm the reported qPCR results (Additional file 5).

The low amounts of recovered viral nucleic acids in this assay require initial transfection of viral DNA as previously published [14]. It must be noted therefore, that the viral RNA is thus not being delivered to the cell as in normal infection and reverse transcription is not taking place. Nevertheless our results suggest an association of REAF with viral nucleic acids. Since the restriction acts in the early phase of replication we hypothesise that REAF interacts with RNA:DNA intermediates or dsDNA products of HIV reverse transcription, either directly or indirectly targeting them for degradation.

### REAF is downregulated in response to HIV-1 infection and restriction requires reverse transcription

**Figure 4:**
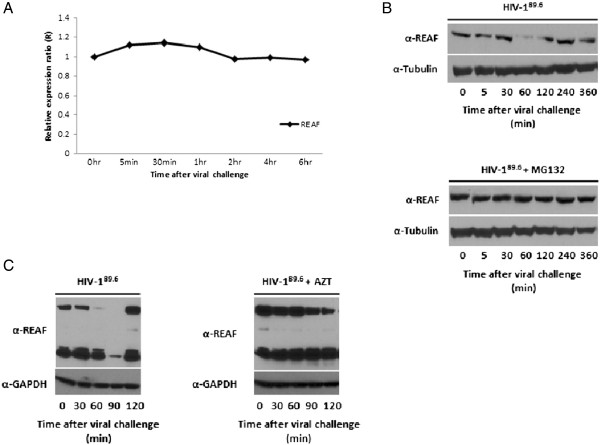
**REAF is downregulated in response to HIV-1 infection and restriction requires reverse transcription. (A)** Time course of REAF mRNA following challenge with HIV-1^89.6^. qPCR results from analysis of cDNA are normalised to β-actin mRNA levels. **(B)** REAF protein levels are downregulated following challenge with HIV-1^89.6^. Treatment with MG132 (10 μM) prevents the degradation of REAF protein. Tubulin is included as a loading control. Only the lower band of REAF (80 kDa) is shown – not all bands are detected in all experiments. **(C)** Following treatment with AZT (100 nM), challenge with HIV-1^89.6^ no longer causes depletion of REAF at 1 hr pi. GAPDH is included as a loading control.

One characteristic of viral restriction factors is that viruses evolve a means of bypassing them. We tested whether HIV infection affected the levels of REAF expression in cells. RNA was extracted from HeLa-CD4 cells at various time points after challenge with HIV-1^89.6^. RT-qPCR of these samples showed that REAF mRNA is unaffected following viral infection (Figure 4A). REAF protein levels however, decrease in the initial hours following challenge with HIV-1, recovering to pre-infection levels by 2 hours (Figure 4B and C). The level of REAF protein does not decrease in the presence of the proteasome inhibitor MG132 suggesting that this protein is targeted to conventional proteosomal degradation (Figure 4B). It is possible that the down modulation observed is mediated by the virus in an attempt to facilitate the establishment of infection, similar to that seen for APOBEC3G and SAMHD1 [15,16]. Thus HIV-1 may have evolved a means of mitigating the effects of REAF using cellular proteosomal degradation pathways. Alternatively the degradation of REAF may be a by-product of the restriction process and occur after it associates with its viral target as a part of the normal cellular process of viral restriction. Further experimentation is required to determine the trigger for the loss of REAF post viral challenge.

To further characterise this virus-REAF interplay we investigated whether reverse transcription itself plays a role. A similar time course of infection was performed on HeLa-CD4 cells that had been pre-treated with AZT (Figure 4C). In contrast to untreated cells, REAF was not degraded in the presence of AZT, suggesting that initiation of reverse transcription is required to trigger degradation of REAF. We hypothesise that REAF interacts either with the products of reverse transcription (RNA:DNA intermediates or dsDNA) directly or indirectly to restrict replication and is degraded in the proteasome.

## Discussion

Our results demonstrate that REAF is a new potent anti-HIV/SIV restriction factor. The timing of both the restriction to viral replication and the virus’ potential counterattack supports our model that REAF acts upon a viral nucleic acid target at an early post-entry stage of infection, before the viral genome reaches the nucleus.

The phenotype of a restriction factor is most readily observed for REAF following its knockdown in HeLa-CD4 cells by siRNA. These experiments consistently show >50 fold rescue of viral titre for HIV-1 and even greater for HIV-2. Interestingly, REAF is also able to act against other non-human retroviruses, suggesting a common viral target. This restriction is confirmed by over-expression of REAF-EGFP, resulting in a decrease in the amount of both FFU/ml and HIV-1 DNA amplified by qPCR compared with cells transfected with empty vector (pEGFP-C3) and challenged with virus. REAF is similar to already identified restriction factors TRIM5α, APOBECs and SAMHD1 because it acts at an early post-entry stage of infection. An increase in the earliest detectable form of RT products (−sssDNA) is seen within the first 1–2 hours following viral challenge in cells down modulated for REAF, and this difference grows steadily with time. The accumulation of full length HIV-1 DNA transcripts results in higher levels of integration, which will impact the ability of the virus to spread more quickly through multiple rounds of infection. Of course we cannot rule out that REAF may additionally block a step after reverse transcription. The apparent ability of REAF to bind DNA, along with our results showing higher rescue of infected cells relative to RT products in REAF knockdown cells, would be consistent with this hypothesis.

The viral target itself probably involves nucleic acid recognition: indeed we demonstrate that REAF associates with viral nucleic acids. However we cannot be sure whether the interaction is direct or indirect. The interaction with nucleic acids suggests that the surveillance mechanism may have implications for the invasion of cells by any RNA virus.

A characteristic of restriction factors of HIV-1 is that viruses evolve to mitigate their effects. Here we observed that REAF is destroyed after viral infection. As early as 1 hour after viral challenge, REAF protein levels are depleted in HeLa-CD4 cells, yet REAF mRNA levels remain constant throughout infection. Depletion of REAF is prevented in the presence of proteasomal inhibitor suggesting that REAF is specifically targeted for degradation through cellular proteasomal pathways. The early time frame for this virally induced degradation coincides with the point in the viral life cycle restricted by REAF. It is possible that HIV-1 targets cellular REAF precisely to prevent inhibition of its critical initiation of reverse transcription. The hypothesis that REAF targets viral replication after the initiation of reverse transcription is supported by the abrogation of REAF protein down modulation in the presence of the AZT. However we cannot exclude that REAF is being destroyed by a cellular programme activated after it recognises its target as part of the restriction process.

In recent years, several cellular factors with the ability to suppress HIV-1 replication early in the viral life cycle have been described. It would therefore not be surprising if the virus has evolved to counteract this onslaught. Studies so far have shown that APOBEC and tetherin, as well as the recently discovered SAMHD1, are antagonised by viral proteins HIV-1 Vif, Vpu and HIV-2 Vpx respectively [9,15,17]. The potential of a viral antagonist for REAF has not yet been determined. The destruction of REAF is similar to that of SAMHD1 which is targeted for cellular proteasomal degradation by HIV-2 Vpx [15].

## Conclusions

The mechanism of REAF anti-viral activity shares similarities with known restriction factors APOBEC, tetherin and SAMHD1 and is an important new addition to the cellular armoury against viral infection. It is vital to understand the interaction of HIV and innate cellular restriction factors as they represent a relatively untapped source of therapeutic potential. Small molecule pharmaceuticals capable of regulating a pathway of interest to enhance the interaction between a viral protein and its cellular antagonist may be a valuable approach.

## Methods

### Cells

Culture of HEK 293 T, HeLa-CD4 and C8166 cells and their optimal culture conditions have been described previously [12,18].

### Plasmids and virus production

The REAF-EGFP expression plasmid was generated by PCR amplifying the open reading frame from HeLa-CD4 cDNA. The vector used was pEGFP-C3 (Clontech). The infectious molecular clone for HIV-1^89.6^ was obtained from the Centre for AIDS Research (NIBSC, UK). Plasmid construct HIV-1^89.6Δenv^ was generated from the HIV-1^89.6^ molecular clone using overlap extension PCR. Clones were confirmed by plasmid sequencing (Source BioScience). Primers for all constructs are available upon request. Virus stocks were prepared from infectious full-length or pseudotyped HIV clones by polyethylenimine (Polysciences) or Lipofectamine 2000 (Invitrogen) transfection of HEK 293 T cells. HIV-2^CBL-23^ and SIV stocks were grown in C8166 cells.

### Preparation of protein lysates

Prior to analysis on SDS-PAGE gels, cell pellets were lysed in ice cold lysis buffer (50 mM Tris pH 7.5, 150 mM NaCl, 1% NP-40, 0.25% sodium deoxycholate) containing protease inhibitor cocktail (Roche Applied Sciences) and 5 mM of sodium fluoride, β-glycerophosphate and sodium orthovanadate.

### Western blot and immunoprecipitations

SDS-PAGE separated proteins, immobilised on nitrocellulose membrane (Novex) were detected with the primary rabbit polyclonal antibody against REAF (RbpAb-RPRD2), rabbit pAb-GAPDH, rabbit pAb-PABP or rat pAb-tubulin (Abcam) followed by corresponding horseradish peroxidise-conjugated goat α-rabbit/rat antibody (Abcam). Proteins were visualised using a chemiluminescence kit (ECL/ECL Prime, GE Healthcare).

### Oligo (dT) immunoprecipitation

Prior to oligo (dT) addition, control samples were treated with DNase (10U) or increasing amounts of an RNaseA/H cocktail (250-2500U RNaseA, 20U RNaseH) and incubated at 37°C for 15 min. DNase (DNA-*free*™ Kit; Invitrogen) was physically removed from the appropriate sample according to manufacturer’s instructions. Ribonucleoprotein properties of REAF were then investigated by mixing lysates prepared as above with oligo (dT) magnetic beads (Invitrogen). After 15 min at 4°C the oligo (dT)-protein complexes were washed extensively with lysis buffer and TE. The complex was eluted into 1× PAGE loading buffer and analysed by Western blot.

### Viral RNA IP and RT-qPCR analysis

Immunoprecipitation of viral RNA was performed as previously reported with slight modifications [14]. Briefly, HEK 293 T cells were transfected with either pEGFP-C3 or REAF-EGFP and HIV-1^89.6^ molecular clone DNA using Lipofectamine 2000 (Invitrogen). Cells were washed 2× with ice cold PBS then 2× with ice cold swelling buffer (25 mM HEPES, 1.5 mM MgCl_2_, 85 mM KCl, pH 8.0) and lysed in ice cold lysis buffer (25 mM HEPES, 1.5 mM MgCl_2_, 85 mM KCl, 0.02% NP-40, 1% Triton X-100, pH 8.0) containing RNaseOUT (500U/ml, Invitrogen), protease inhibitor cocktail (Roche Applied Sciences) and 10 mM of sodium fluoride, sodium pyrophosphate, β-glycerophosphate, and sodium orthovanadate. After centrifugation, the lysate was incubated with 2U of DNase (Ambion) for 5 min at 37°C and pre-cleared with Protein A/G-coupled rabbit IgG beads for 30 min at 4°C. One tenth of the volume was kept for total cellular RNA input, one tenth for Western blotting input and the rest mixed with Protein A/G-coupled REAF Ab beads and incubated for 1 hr at 4°C. The bead bound complexes were washed 2× with lysis buffer, 2× with wash buffer (25 mM HEPES, 1.5 mM MgCl_2_, 85 mM KCl, 0.25% Triton X-100, pH 8.0) containing 0.1U/ml RNaseOUT, 2× in wash buffer supplemented with 0.4 M NaCl followed by 2 washes with wash buffer supplemented with 0.1U/ml RNaseOUT and 0.1% v/v SDS. An aliquot of the complex was kept for Western blotting while the rest was incubated with DNase for 10 min followed by two phenol:chloroform:isoamyl alcohol (50:49:1) and one chloroform extraction. After precipitation with ethanol and glycogen carrier (Ambion), the RNA pellet was resuspended in DEPC-treated water and reverse transcribed using Superscript III First Strand Synthesis System (Invitrogen) according to manufacturer’s instructions. The resulting cDNA was used in qPCR analysis with primers for RU5 and late HIV-1 DNA as previously described [19]. Primers are available upon request. This qPCR was repeated in a standard PCR machine for a non-saturating number of amplification cycles. These PCR products were resolved on a 4% agarose gel for visual confirmation.

### siRNA transfection and infection with replication competent virus

HeLa-CD4 cells were seeded at 2.5 × 10^4^ cells/well in 24-well plates. siRNA transfection (30nM) was performed using HiPerfect (QIAGEN) according to the manufacturer’s instructions. 72 hr after siRNA transfection, cells were challenged with virus (MOI 0.2) for up to 5 hr. Infection was assessed up to 48 hr by intracellular p24 staining or qPCR analysis. When required, reverse transcriptase inhibitor AZT (100 μM) and proteasomal inhibitor MG132 (10 μM) were added 1 hr before infection.

### In situ immunostaining for p24 antigen

Infected cells were fixed with cold (−20°C) methanol:acetone (1:1), washed with PBS then immunostained for p24 using mouse anti-HIV-1 p24 monoclonal antibodies EVA365 and 366 (NIBSC, UK) (1:50) or anti-SIV p27 monoclonal antibodies (1:500) (to detect SIV infected cells), as previously described [20]. Infected cells were blue (regarded as foci of infection (FFU/ml)) and quantitated by light microscopy.

### First round Alu-PCR

DNA was extracted at various time points after infection with the QIAamp DNA Blood Mini Kit (QIAGEN). Integrated HIV-1 DNA was measured by nested PCR, as previously described [19].

### qPCR for HIV-1 DNA

The isolated DNA was subjected to qPCR to determine the number of early (negative strand strong stop DNA, -sssDNA), RU5 and late (*gag*) transcripts, normalised for cell number by genomic GAPDH as previously described [19].

### cDNA synthesis and mRNA analysis

Total HeLa-CD4 RNA was extracted using an RNeasy Plant Mini Kit (QIAGEN) and cDNA was synthesised with Superscript III First Strand Synthesis System (Invitrogen), according to manufacturer’s instructions. The cDNA produced was subjected to qPCR as previously described [19]. Primer sequences used to determine REAF mRNA levels are available upon request.

## Competing interests

The authors declare no competing interests.

## Authors’ contributions

KMM and BWO performed the experiments with assistance from CP, NMMO and EO’S for molecular analyses and infection assays. KMM and ÁMcK conceived the project and wrote the paper. All authors read and approved the final manuscript.

## Supplementary Material

Additional file 1 **Validation of REAF siRNA.** (A) The four siRNAs from the original screening pool were individually tested at 30nM using the same protocol as the siRNA screen [6]. For REAF, 3/4 siRNAs enhanced virus replication. (B) Target sequence of the 4 REAF siRNAs tested. REAF 4 siRNA was used in all subsequent knockdown experiments except where stated. Click here for file

Additional file 2 **siRNA knockdown of REAF rescues viral replication.** siRNA knockdown of REAF rescues infection of HeLa-CD4 cells by HIV-1^89.6^, HIV-2^CBL-23^, SIV^AGM^ (African Green Monkey; TYO-1), SIV^MAC^ (Macaque; 32H) and SIV^SM^ (Sooty Mangabey; B670) infection compared with a non-targeting siRNA control (CB). Raw data is shown as FFU/ml and are mean ± s.d. of a representative experiment performed in duplicate. Click here for file

Additional file 3 **Validation of REAF 3’UTR siRNA.** (A) Western blot of HeLa-CD4 cell lysate following REAF 3’UTR siRNA knockdown compared with CB control. Only the 80 and 220 kDa bands are detectable. GAPDH (36 kDa) is added as a loading control. (B) Target sequence of REAF 3’UTR siRNA. Click here for file

Additional file 4 **Transfection efficiency of REAF-EGFP compared to pEGFP-C3.** HeLa-CD4 cells transfected with REAF-EGFP or pEGFP-C3 empty vector. Cells were analysed by immunofluorescence 24 hr post transfection. Click here for file

Additional file 5 **REAF associates with viral nucleic acids.** RU5 and late HIV-1 DNA were amplified by standard RT-PCR from RNA isolated from viral RNA IP. PCR program was terminated at a non-saturating amplification cycle and reaction products were run on a 4% agarose gel for visual confirmation. RU5 and late PCR products are detectable in input samples (lanes 1 and 3) and following IP of either endogenous (lane 9) or exogenous (REAF-EGFP) (lane 11) REAF with α-REAF antibody, but not after IP with IgG alone (lanes 5 and 7). Click here for file
