# Supplementary material for: Novel restriction factor RNA-associated early-stage anti-viral factor (REAF) inhibits human and simian immunodeficiency viruses
Source: Retrovirology. 2014 Jan 10;11:3. doi: 10.1186/1742-4690-11-3 (PMC3895926; doi:10.1186/1742-4690-11-3)
Supplement: Additional file 1 — Validation of REAF siRNA. (A) The four siRNAs from the original screening pool were individually tested at 30nM using the same protocol as the siRNA screen [6]. For REAF, 3/4 siRNAs enhanced virus replication. (B) Target sequence of the 4 REAF siRNAs tested. REAF 4 siRNA was used in all subsequent knockdown experiments except where stated. [file 1742-4690-11-3-S1.pdf]

AF1

A

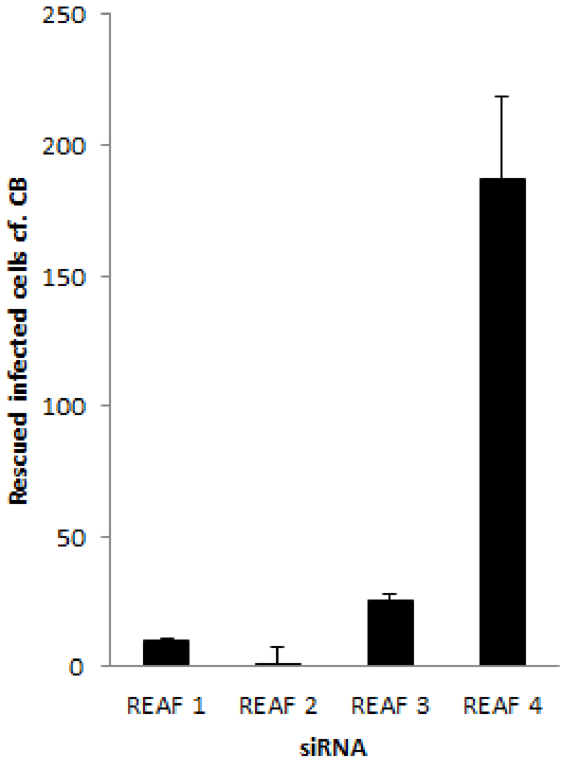

B

siRNA	Target sequence
REAF 1	AGCCGGAGTGGTATAATCTTA
REAF 2	ACCGGTCACCATCACCGAGTA
REAF 3	CACGTCGACGATTGAATTTAA
REAF 4	CACGTAAGCCCTCAGATGATA
