# Supplementary figures and images for: Novel restriction factor RNA-associated early-stage anti-viral factor (REAF) inhibits human and simian immunodeficiency viruses
Source: Retrovirology. 2014 Jan 10;11:3. doi: 10.1186/1742-4690-11-3 (PMC3895926; doi:10.1186/1742-4690-11-3)

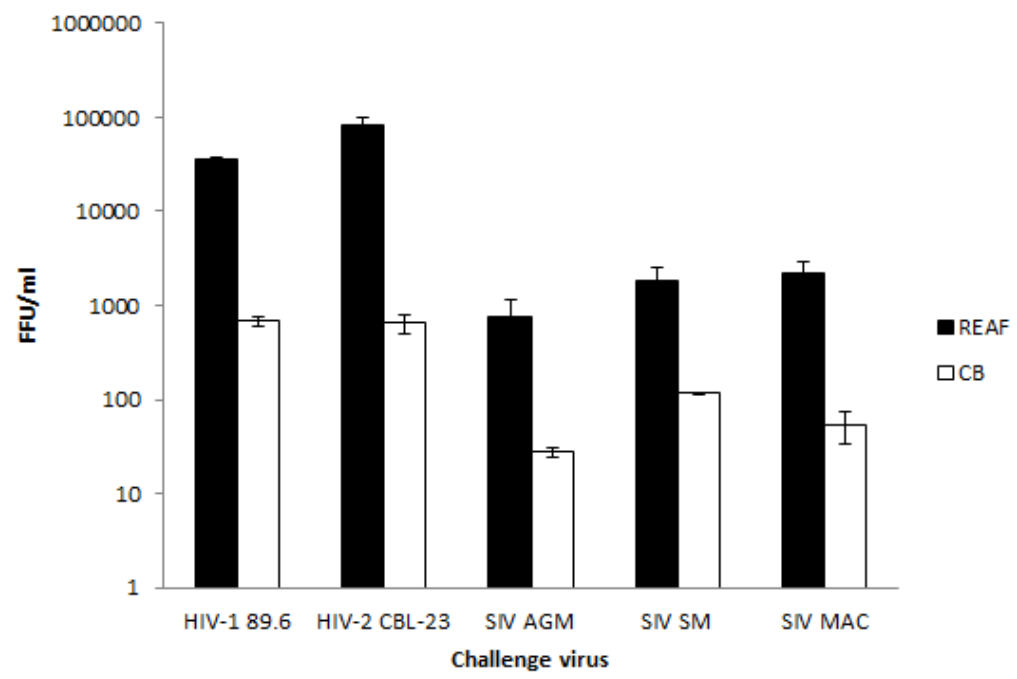

Supplement: Additional file 2 — siRNA knockdown of REAF rescues viral replication. siRNA knockdown of REAF rescues infection of HeLa-CD4 cells by HIV-189.6, HIV-2CBL-23, SIVAGM (African Green Monkey; TYO-1), SIVMAC (Macaque; 32H) and SIVSM (Sooty Mangabey; B670) infection compared with a non-targeting siRNA control (CB). Raw data is shown as FFU/ml and are mean ± s.d. of a representative experiment performed in duplicate. [file 1742-4690-11-3-S2.pdf]

A

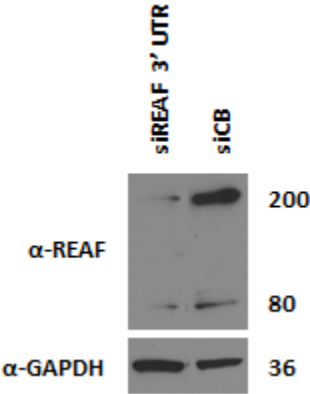

B

| siRNA      | Target sequence       |
|------------|-----------------------|
| REAF 3'UTR | GGGCGGGATAATTGTCCTCAA |

Supplement: Additional file 3 — Validation of REAF 3’UTR siRNA. (A) Western blot of HeLa-CD4 cell lysate following REAF 3’UTR siRNA knockdown compared with CB control. Only the 80 and 220 kDa bands are detectable. GAPDH (36 kDa) is added as a loading control. (B) Target sequence of REAF 3’UTR siRNA. [file 1742-4690-11-3-S3.pdf]

AF4

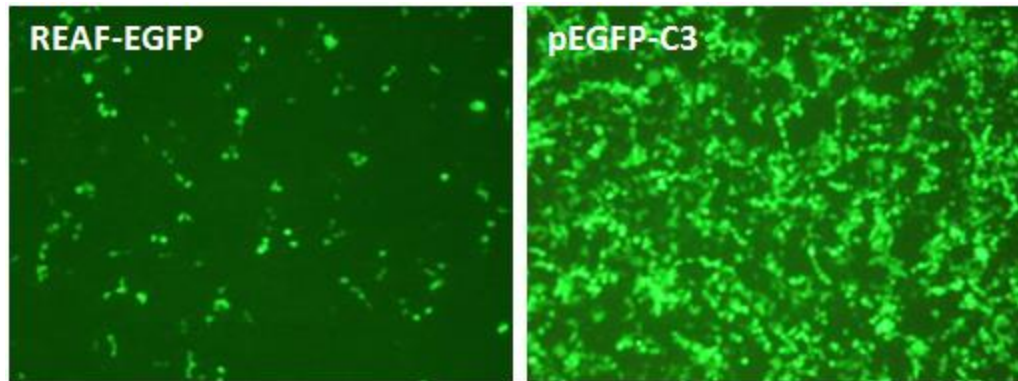

Supplement: Additional file 4 — Transfection efficiency of REAF-EGFP compared to pEGFP-C3. HeLa-CD4 cells transfected with REAF-EGFP or pEGFP-C3 empty vector. Cells were analysed by immunofluorescence 24 hr post transfection. [file 1742-4690-11-3-S4.pdf]

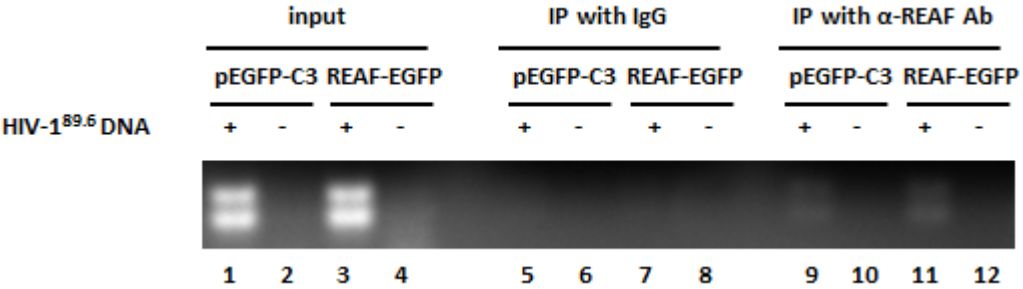

Supplement: Additional file 5 — REAF associates with viral nucleic acids. RU5 and late HIV-1 DNA were amplified by standard RT-PCR from RNA isolated from viral RNA IP. PCR program was terminated at a non-saturating amplification cycle and reaction products were run on a 4% agarose gel for visual confirmation. RU5 and late PCR products are detectable in input samples (lanes 1 and 3) and following IP of either endogenous (lane 9) or exogenous (REAF-EGFP) (lane 11) REAF with α-REAF antibody, but not after IP with IgG alone (lanes 5 and 7). [file 1742-4690-11-3-S5.pdf]
